# Gustatory-Visual Interaction in Human Brain Cortex: fNIRS Study

**DOI:** 10.3390/brainsci15010092

**Published:** 2025-01-19

**Authors:** Karolina Jezierska, Aneta Cymbaluk-Płoska, Justyna Zaleska, Wojciech Podraza

**Affiliations:** 1 Department of Medical Physics, Pomeranian Medical University in Szczecin, ul. Ku Słońcu 13, 71-073 Szczecin, Poland; podrazaw5@gmail.com; 2 Department of Reconstructive Surgery and Gynecological Oncology, Pomeranian Medical University in Szczecin, Al. Powstańców Wielkopolskich 72, 70-111 Szczecin, Poland; aneta.cymbaluk.ploska@pum.edu.pl; 3 Department of Gynecology and Obstetrics, Pomeranian Medical University in Szczecin, Al. Powstańców Wielkopolskich 72, 70-111 Szczecin, Poland; j.zaleska2@gmail.com

**Keywords:** fNIRS, taste stimulation, human brain cortex, gustatory-visual interaction

## Abstract

Background: Many studies, for example, on taste-visual dissonance, have shown that the influence of the visual cortex on taste sensation is enormous. The presented work aims to investigate, using fNIRS, whether a taste stimulus, in this case, the taste of bitter, also causes stimulation of the visual cortex in the fNIRS study. Methods: fNIRS was used to examine 51 participants (204 examinations, 9996 records), collecting signals from the left hemisphere. Differences between the maximum and minimum changes in oxyHb concentrations (ΔoxyHb) for the areas of the brain cortex considered responsible for recording visual and gustatory signals were analyzed. Protocols I, II, III, and IV—activation with distillate water, coffee with lower concentration, reference (no stimulation), and coffee with higher concentration, respectively, were used. Results: We recorded high signals for teste activation on channels covering the gustatory cortex, which confirms the correctness of the choice of research method. As expected, a significant statistical difference was observed between protocols I, II, and IV and reference III (without stimulation). What seems important is the fact that we also received high signals for the channels 45–49, which cover the visual cortex. The statistical analysis shows no differences between protocols I, II, and IV (different taste activation—water, coffee A, and coffee B) for specific channels for analyzing regions of interest. As a result of the analysis of the correlation between the subjective bitterness assessment solutions and the signal ΔoxyHb height, it was observed that a statistically significant correlation, although weak, occurs only for 14 and gustatory channels, only for coffee with a higher concentration. Additionally, the only statistically significant difference between women and men was observed in Protocol I (water), where the ΔoxyHb signal was twice as high in women compared to men. Conclusions: In conclusion, we can clearly state that the senses of sight and taste work closely together. Moreover, this cooperation is not one-sided: while visual activation influences taste perception, interestingly, a taste stimulus can also generate a hemodynamic response, activating the visual cortex.

## 1. Introduction

In most life situations, the senses cooperate, providing consistent information about the world around us, which is of great importance for the survival of our species. For example, beautiful flowers usually smell pleasant, while things containing toxic substances in nature are generally bitter and repel us with their appearance. Often unconsciously, based on impressions provided by one sense, we “create” information for ourselves, completing the whole picture with information we cannot access at a given moment, which other senses should provide. For example, we imagine his appearance based on a telephone conversation (e.g., the timbre of the interlocutor’s voice). Cooperation between sight and taste, which is the focus of the proposed manuscript, is robust. For example, red-colored drinks are often associated with the flavors of specific fruits, such as strawberries or cherries. Our experiences teach us predictable connections between food’s appearance, smell, taste, and nutritional value. These associations help us construct a conceptual model of the foods we consume, which is often leveraged in food product advertising.

Many studies, for example, on taste-visual dissonance, have shown that the influence of the visual cortex on taste sensation is enormous [1,2,3,4,5]. Research has demonstrated that the appearance, particularly the color, of food products influences their taste perception. These findings underscore the interplay between visual cues and gustatory perception, highlighting the critical role of visual stimuli in shaping sensory experiences. Thus, we can say that we “eat with our eyes”, as the appearance of a dish is undoubtedly crucial to us and can trigger activation in the gustatory cortex [6,7].

Is the reverse true? Can a taste stimulus cause a response in the visual cortex? When we eat or drink, do we unconsciously trigger visual impressions in our brains? The authors did not find any studies in the literature answering this question, and the only way to answer it today is through functional brain research such as fMRI or fNIRS.

fMRI is a well-known method and allows the examination of the entire brain, but it requires enormous equipment, space, and personnel requirements. Hence, it is a relatively complex and expensive test [8,9,10,11,12]. A relatively new and engaging method of functional brain research is fNIRS—functional near-infrared spectroscopy. This method, used in neurology and neurobiology, uses near-infrared spectroscopy for functional neuroimaging, including the study of the response of the cerebral cortex to taste stimuli [13,14]. Using fNIRS, brain activity is measured by hemodynamic responses related to neuronal behavior [15]. This non-invasive test allows you to determine, based on IR absorption by hemoglobin and oxyhemoglobin, which areas of the cerebral cortex are characterized by a greater demand for oxygen and nutrients after a specific stimulus [16]. Although this very sophisticated method is sensitive to changes in oxyHb levels lower than 10^−3^ mmol/L (1 μM), its undoubted disadvantage is the possibility of examining only the cerebral cortex (up to 1.5 cm) and the inability to refer to the anatomy as precisely as in the case of fMRI [16,17].

In the described method, the light sources—emitters are most often LEDs or lasers generating NIR radiation with a precisely defined wavelength λ, in the range of 700–900 nm, which is then recorded by photodetectors placed at a certain distance after passing through the tissue. The diode–detector system creates a channel. The beam’s absorbance depends on the Hb and HbO concentration in the blood in the examined tissue volume. Greater oxygen demand and consumption are observed during the activation of specific areas of the cerebral cortex by a nerve signal resulting from stimulation with a particular stimulus or during a task involving specific processes (e.g., movement). This causes the increased local blood flow in the cerebral cortex, which is called the hemodynamic response or, most commonly, neurovascular coupling. In the first stage, a short-term decrease in blood oxygenation is observed. The inflow of oxygenated blood relatively quickly compensates for this decrease, but the total amount of oxygen transported to the active brain area exceeds its metabolic capacity. This results in a local increase in the amount of oxygenated blood and a decrease in the amount of deoxygenated blood. The location of the channels is strictly dictated by the location of the cortical areas to be observed/stimulated in a given experiment. fNIRS measures changes in oxyhemoglobin (OxyHb) and deoxyhemoglobin (DeoxyHb) levels, reflecting oxygen demand in active brain regions. When the stimulus is no longer novel or relevant, the demand for oxygen in activated regions decreases, leading to a gradual signal decay, which is well observed in Figure 3.

This study aims to determine whether a taste stimulus, the bitter/coffee taste, induces activation of the visual cortex in an fNIRS investigation.

## 2. Materials and Methods

**Figure 1:**
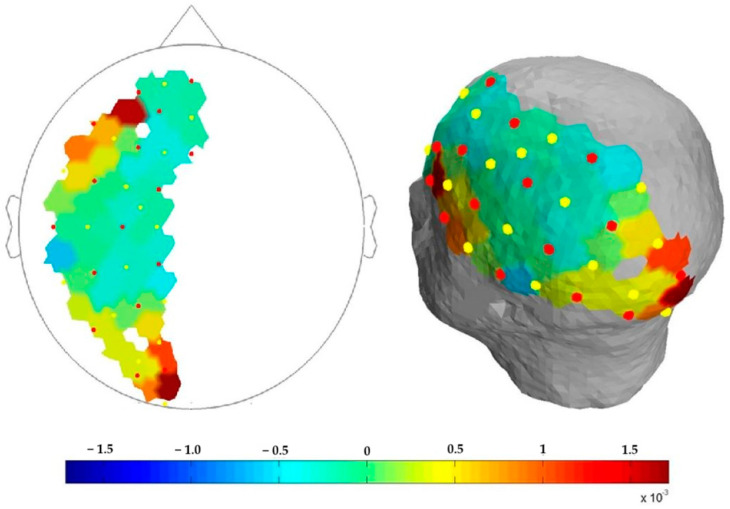
Diagram of optodes arrangement on the head surface. The scale shows the change in oxyhemoglobin concentration [mmol/L]; Red dots indicate light sources, yellow dots represent light detectors.

The study was conducted in compliance with the decision of the local bioethics committee. All 51 volunteers provided written informed consent and were fully briefed on the study’s purpose and procedures. fNIRS spectroscope (NIRScout, NIRx Medical Technologies LLC, Glean Head, NY, USA) was used to record near-infrared brain signals. Light from eight dual-wavelength LED light sources (wavelengths 760 and 850 nm) was detected by avalanche photodiode (APD) optodes placed 3 cm from the emitters. Optodes were placed to cover the left hemisphere (mostly the dominant hemisphere), as shown in Figure 1.

All measurements were recorded using NIRStar 15.0 (NIRx Medizintechnik GmbH, Berlin, Germany) software. Signal intensity was calibrated and validated for each channel before data collection, and a low-pass filter was applied during data acquisition. Participants were instructed to remain seated with their eyes closed for the study.

**Figure 2:**
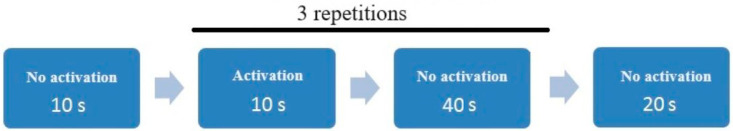
Scheme of study. Activation is understood as stimulation of the cerebral cortex with 0.5 mL of water, coffee A, or coffee B, or no stimulation for protocol III (reference one).

Four protocols, each with the same scheme of study, were used to carry out the research project for every participant in the same order, in two-minute intervals (Figure 2).

During the measurements, we were very strict about ensuring the patients did not perform any unnecessary actions or movements. The cap was attached directly to the chest, bypassing the mandible and allowing us to eliminate jaw movement/swallowing artifacts. Protocol I, II, and IV—activation with distillate water, coffee A, and coffee B, respectively. In all protocols on the sound signal, the participants opened their mouths, and 0.5 mL (with the pipette) of substance was dropped (there were three repetitions). Protocol III’s study scheme was identical; however, no activation (reference measurement) was performed.

2% (coffee B) and 0.4% (coffee A) solutions of instant coffee were prepared. For coffee B, 2 g of instant coffee was accurately weighed and dissolved in 98 g of distilled water. It was 0.4 g of coffee and 99.6 g of water for coffee B. The water was brought to room temperature before adding the coffee to ensure uniform dissolution conditions. The mixture was stirred manually using a glass rod for approximately 1 min until the coffee was dissolved entirely. The resulting solution was stored in a tightly sealed container and used immediately in the subsequent analysis.

During the experiment, there was a short break of several minutes between trials to allow participants to rinse their mouths with distilled water. This ensured that any residual taste from the previous sample did not interfere with the subsequent evaluations.

Study participants completed a short questionnaire in which, in addition to data on age, gender, height, handedness, and weight, they assessed the bitterness of both coffee solutions and declared the amount of coffee they drank per day.

The data acquisition parameters were consistent across all protocols. All data were analyzed using MATLAB-based NIRSLab 15.0. The recordings were band-pass filtered with a high cutoff frequency of 0.2 Hz and a low cutoff frequency of 0.01 Hz. The typical spectrum for oxyHb and deoxy-Hb used in the analyses was created based on the manufacturer’s recommendations. The final data for each participant was the average of three repetitions.

After preliminary visual analysis, the highest signals were recorded for two channels, 14 and 48. The first corresponds to the location of the cortical taste center and the second to the visual cortex. Based on our observation and the literature to investigate gustatory-visual interaction, we analyzed the data for the whole cortical taste center (channels 14, 21, 28, 29, and 30) [13,17], visual cortex (45, 46, 47, 48, and 49) [18] and additionally separately 14, and 48 channel. fOLD v2.2 software was used to locate the optodes.

Data regarding the difference between the maximum and minimum changes in oxyHb concentrations (ΔoxyHb) were collected and analyzed (in summary, 51 volunteers, 204 examinations, 9 996 records).

To assess the effect of the taste activation for water and coffee on the change in oxyHb concentration in the cortex, data obtained from protocols I, II, and IV were compared with data from protocol III (reference). Data from protocols I, II, and IV were compared to evaluate the influence of water, coffee A, and coffee B on oxyHb.

Studying the cortical response to taste stimuli requires eliminating potential artifacts associated with jaw movement. Additional experiments were conducted to evaluate the potential impact of mouth opening on the signal. We analyzed the effect of mouth opening on recorded signals (protocol” jaw movement”) to rule out the possibility that observed changes in cortical response were due to movement rather than the taste stimulus.

Data distribution was assessed using the Shapiro-Wilk test. To investigate the differences between the protocols for normally distributed data, the Student’s t-test was used; otherwise, the Mann-Whitney U-test was performed (Dell Inc., One Dell Way, Round Rock, TX 78682, USA, 2016, Dell Statistica [data analysis software system], version 13. software.dell.com; accessed on 1 June 2024). The Bonferroni correction was used because of the multiple comparisons problem, so differences were considered statistically significant for *p* values < 0.0125. Spearman tests were used for correlation analysis. Correlations were considered statistically significant for *p* values < 0.05.

## 3. Results

The study involved 51 healthy volunteers (41 women, 10 men) with a median age of 19 years (18–21 years), mean height of 168 cm (155–190 cm), and mean weight of 65 kg (40–135 kg). Only four women were left-handed. In Protocols I, II, and IV, characteristic reactions dependent on blood oxygenation levels were observed, defined by increased oxyHb concentration and decreased deoxyHb levels across all analyzed channels following stimulation. The increase of ΔoxyHb (difference between max and min oxyHb value) was analyzed up to 15 s after the stimulus for every protocol. The statistical information on ΔoxyHb [mmol/L] values for all protocols, for all males and females, are shown in Table 1, Table 2 and Table 3.

**Figure 3:**
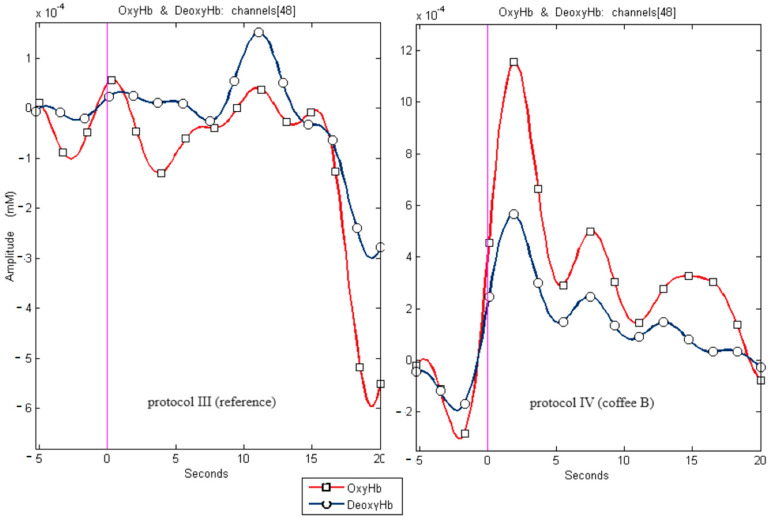
Sample recordings for channel 48 for protocols IV and III (reference) for channel 48. The presented graph illustrates changes in oxyhemoglobin (OxyHb) and deoxyhemoglobin (DeoxyHb) concentrations in channel 48 for two protocols, III (reference) and IV (taste activation). The red line with square markers represents changes in OxyHb concentration over time. The blue line with circular markers illustrates changes in DeoxyHb concentration. The pink vertical line indicates the moment the taste stimulus was administered. Channel 48 corresponds to areas of the brain cortex responsible for registering visual stimuli. The data demonstrate the hemodynamic response of the cerebral cortex to stimuli, with an apparent increase in OxyHb and a decrease in DeoxyHb during the activation period. Differences in signals between the reference and activation protocols indicate the activation of specific brain regions in response to the taste stimulus, confirming the interplay between the senses of taste and vision.

In some cases, fewer than 51 results were presented because certain records were disqualified due to artifacts that rendered their evaluation impossible. Sample recordings for channel 48 in Protocols IV and III are shown in Figure 3.

**Figure 4:**
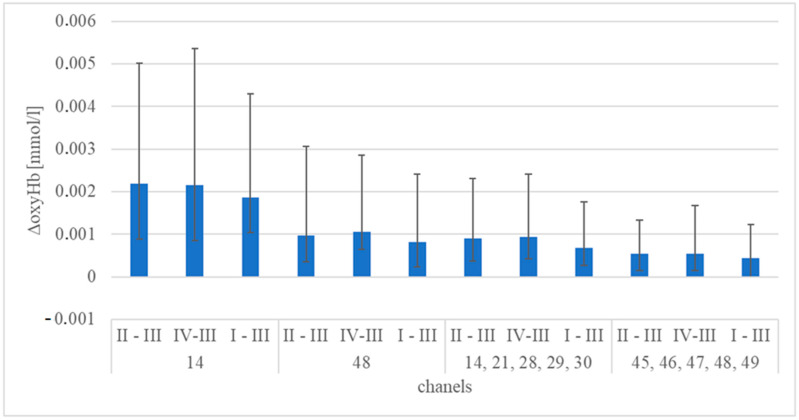
Difference between the median of ΔoxyHb for protocols I, II, and IV and the median for reference protocol III for all participants, with error bars representing the respective quartiles: Q1 (lower error bound) and Q3 (upper error bound).

Figure 4 and Table 4 and Table 5 present the statistical comparison results of median ΔoxyHb values for Protocols I, II, III, and IV across all analyzed channels (for all participants).

The results of the statistical comparison of medians ΔoxyHb for all channels and all protocols (for all participants) are presented in Table 6, Table 7 and Table 8.

The correlations were examined between the amount of coffee consumed (ACC), the bitterness assessment solutions (bitterness), and the signal height for individual channels. The results of Spearman’s rank correlation test are collected in Table 9.

Differences in signal height between females and men for all protocols and all channels were also examined (Mann-Whitney U-test). The only statistically significant differences for ΔoxyHb were obtained for channel 14, for protocol I (water). The median/mean for females and males was 0.00214/0.00226 and 0.00128/0.00134, respectively, concerning reference protocol (I–III) 0.00200/0.00199 and 0.00108/0.00111 (see Table 2 and Table 3).

Table 10 shows the results of additional measurements intended to exclude the influence of jaw movement on stimulation in the visual cortex. Statistical analysis revealed no significant differences between the reference (III) and the group where participants only opened their mouths (*p* > 0.05).

## 4. Discussion

Taste is a complex sense essential for species to differentiate between harmful and nutritious foods [19]. Because taste governs food acceptability, it has a critical role in human survival, and, in general, it can affect health conditions [20].

Numerous studies in the human brain mapping cortex field prove that the reception and analysis of stimuli, including gustatory ones, reaching us from various senses is not one-track. As a result of the conducted research, including functional brain research, it turns out that information about the external and internal world (our body) in the cerebral cortex is mixed [21,22,23]. Until recently, it seemed that we knew perfectly well which pathways and to which places in the cerebral cortex signals from specific senses reach. It turns out, however, that it is not so obvious. Hence, it is still a research topic in neurology, neurocognitive science, and physiology. The topic of the presented work focuses on the taste stimulus. It would seem that this simple stimulus, for example, a bitter taste, would induce hemodynamic activity only in the gustatory cortex, too.

In our previous work on the hemodynamic response of the cerebral cortex after sour taste activation, we initially noticed that the gustatory cortex is not the only place in our cerebral cortex where a taste stimulus generates an increased hemodynamic response [24]. Numerous publications also show the influence of the sense of sight on the perception of taste, which seems quite intuitive. However, the authors found no studies confirming that a taste stimulus can also generate activity in the cerebral cortex in places generally considered responsible only for the reception of visual stimuli. Analyzing the available literature, we can conclude that both senses cooperate closely, so it seems highly probable.

Analysis of over 200 fNIRS recordings has unequivocally confirmed that using taste stimuli, such as water or coffee at varying concentrations, consistently elicited a hemodynamic response in the gustatory cortex. We recorded signals for taste activation on channels 14, 21, 28- 30, which confirms the correctness of the choice of research method. We also received high signals for channels 45–49 covering the visual cortex. This may indicate that when tasting various dishes and drinks, we involuntarily and unconsciously create a particular image of the food or drink we consume, perhaps associating it with specific places, situations, or people.

During measurements for protocol III, the patients did not open their mouths. This fact previously was a weakness of the presented work, as there were concerns that the recorded signal for the visual cortex may be the effect of movement artifacts. However, the placement of the cap on the chest, bypassing the jaw, appears to rule out such a possibility. Additionally, the morphology of the recording suggests that we are dealing with stimulation of the visual cortex and not artifacts related to movement. Results from additional measurements confirmed that mouth opening alone did not affect the signal for the visual cortex, supporting the validity of the previously obtained findings (see Table 10). There were no significant differences between protocol III and “jaw movement” (*p* > 0.05).

In future studies, measurements are planned so the participants open their mouths at the right moment during the reference test.

The results indicate that mouth opening does not generate artifacts in the recorded signals (*p* < 0.5). This confirms that the observed cortical activity in the group receiving the coffee stimulus was due to the specific taste stimulus and not the movement of opening the mouth. Additional control experiments were crucial in confirming the validity of the primary experiment’s results.

Figure 4 shows the height of the ΔoxyHb, which confirms the methodology protocol’s correctness. The highest values were recorded for channels 14 and 48, representing gustatory channels (14, 21, 28–30) and visual channels (45–49), respectively. The statistical analysis presented in Table 4 and Table 5 shows no statistical differences between protocols I, II, and IV (different taste activation—water, coffee A, and coffee B) for specific channels. The lack of difference in the hemodynamic response to the taste stimulus of pure water and coffee measured by the objective fNIRS method leads to the conclusion that this method is unsuitable for assessing taste and cannot compete with the subjective declared assessment of the examined person.

There is a statistically significant lower signal for reference protocol III (no activation) compared with taste activation, confirming the methodology’s correctness. However, there are some essential differences between the level of signal ΔoxyHb for different channels, which are presented in Table 6, Table 7 and Table 8. What is the most critical signal for channel 14 is always the highest one. When comparing the signal heights between the 48 gustatory and visual channels, they are not always statistically distinguishable. Hence, it can be concluded that the stimulation of these areas was repeatedly comparable. Based on the collected data, we can clearly state that the senses of sight and taste work closely together. Moreover, this cooperation is not one-sided—not only does the appearance of the food (visual impression) affect the perception of taste, which is quite precisely described in the literature, but interestingly, the taste stimulus (in this study, the taste of coffee and water) can generate a hemodynamic response—activity in the visual cortex. This is surprising because there are no reports in the literature on the stimulation of the visual cortex by a taste stimulus. For example, for a bitter taste, many studies indicate a place that is activated by a bitter stimulus: right precentral gyrus [25,26,27,28,29,30,31], left amygdala [26], right thalamus [11,26,29], and there is no mention of the visual cortex. The research requires repetition on a larger group of subjects. It is also interesting how other flavors would affect the hemodynamic response of the cortex, which also requires further research.

As a result (see Table 9) of the analysis of the correlation between the subjective bitterness assessment solutions and the signal ΔoxyHb height, it was observed that a statically significant correlation, although weak, occurs only for 14 and gustatory channels, only for coffee with a higher concentration. The lack of correlation for coffee A may result from the fact that respondents rated the bitterness of coffee at a similar level (less spread of results). No correlation between the subjective bitterness assessment solutions and the signal ΔoxyHb height for visual channels seems logical—a visual image, e.g., a cup of coffee, will probably “appear” activating the visual cortex at a similar level, regardless of how strong the coffee we drink or what kind of taste stimulus we use in general. There was no statistically significant correlation between the declared amount of consumed coffee and the signal ΔoxyHb height.

Based on research by other researchers, the perception of taste seems to be related to gender, which has not been fully proven in this paper [25,32,33,34,35]. We recorded the only statistically significant difference between women and men for protocol I (water), where the ΔoxyHb signal was twice as high for women. This may indicate that women are more sensitive to taste stimuli, which other researchers confirm. However, this relationship could not be observed for coffee. This is likely due to the study’s small number of male participants. The stronger reaction to water might be because water was the first stimulus used in the research and, therefore, caused higher cortical activation in women than in men. This was observed only in channel 14, which exhibits the most potent response in the taste cortex. Further research on this topic is needed, particularly with more male participants.

## 5. Conclusions

In conclusion, the senses of sight and taste work closely together. Moreover, this cooperation is not one-sided—not only does visual activation affect taste perception, but interestingly, the taste stimulus can generate a hemodynamic response—activity in the visual cortex. This suggests that taste stimuli may involuntarily trigger visual associations, potentially reflecting an adaptive mechanism where sensory integration enhances food perception and decision-making.

## Figures and Tables

**Table 1:** The statistical information on ΔoxyHb [mmol/L] for all protocols and all analyzed channels, where *n*—number of cases, Mdn—median, Min—minimum, Max—maximum, Q1—the 25th percentile, Q3—75th percentile, for all participants. Data were not normally distributed.

Channels	Stimulation (Protocol)	n	ΔoxyHb [mmol/L]		
			Mdn	Q1	Q3
14	water (I)	51	0.00201	0.00090	0.00273
48	water (I)	51	0.00118	0.00077	0.00226
14, 21, 28, 29, 30	water (I)	51	0.00081	0.00048	0.00130
45, 46, 47, 48, 49	water (I)	49	0.00067	0.00055	0.00117
14	coffee A (II)	51	0.00235	0.00138	0.00311
48	coffee A (II)	51	0.00132	0.00080	0.00276
14, 21, 28, 29, 30	coffee A (II)	50	0.00104	0.00061	0.00162
45, 46, 47, 48, 49	coffee A (II)	50	0.00076	0.00049	0.00118
14	coffee B (IV)	50	0.00232	0.00138	0.00348
48	coffee B (IV)	51	0.00142	0.00059	0.00247
14, 21, 28, 29, 30	coffee B (IV)	50	0.00108	0.00060	0.00168
45, 46, 47, 48, 49	coffee B (IV)	50	0.00077	0.00051	0.00150
14	reference (III)	51	0.00016	0.00008	0.00028
48	reference (III)	51	0.00036	0.00018	0.00067
14, 21, 28, 29, 30	reference (III)	51	0.00014	0.00008	0.00021
45, 46, 47, 48, 49	reference (III)	50	0.00023	0.00011	0.00038

**Table 2:** The statistical information on ΔoxyHb [mmol/L] for all protocols and all analyzed channels, Mdn—median, Mn—mean, Min—minimum, Max—maximum, Q1—the 25th percentile, Q3—75th percentile, for male participants. * Results for normally distributed data (Mn, Min, Max).

Channels	Stimulation (Protocol)	n	ΔoxyHb [mmol/L]		
			Mdn/Mn	Q1/Min	Q2/Max
14	water (I)	10	0.00134 *	0.00042 *	0.00242 *
48	water (I)	10	0.00108 *	0.00018 *	0.00271 *
14, 21, 28, 29, 30	water (I)	10	0.00074 *	0.00016 *	0.00152 *
45, 46, 47, 48, 49	water (I)	10	0.00077 *	0.00040 *	0.00147 *
14	coffee A (II)	10	0.00177 *	0.00080 *	0.00323 *
48	coffee A (II)	10	0.00121	0.00083	0.00136
14, 21, 28, 29, 30	coffee A (II)	10	0.00063	0.00053	0.00085
45, 46, 47, 48, 49	coffee A (II)	10	0.00092 *	0.00042 *	0.00199 *
14	coffee B (IV)	10	0.00192 *	0.00082 *	0.00358 *
48	coffee B (IV)	10	0.00139 *	0.00053 *	0.00286 *
14, 21, 28, 29, 30	coffee B (IV)	9	0.00096 *	0.00028 *	0.00141 *
45, 46, 47, 48, 49	coffee B (IV)	10	0.00084	0.00063	0.00125
14	reference (III)	10	0.00023 *	0.00002 *	0.00053 *
48	reference (III)	10	0.00046 *	0.00011 *	0.00102 *
14, 21, 28, 29, 30	reference (III)	10	0.00021 *	0.00000 *	0.00060 *
45, 46, 47, 48, 49	reference (III)	10	0.00022	0.00012	0.00038

**Table 3:** The statistical information on ΔoxyHb [mmol/L] for all protocols and all analyzed channels, where n—number of cases, Mdn—median, Mn—mean, Min—minimum, Max—maximum, Q1—the 25th percentile, Q3—75th percentile, for female participants. * Results for normally distributed data (Mn, Min, Max).

Channels	Stimulation (Protocol)	n	ΔoxyHb [mmol/L]		
			Mdn/Mn	Q1/Min	Q2/Max
14	water (I)	41	0.00214	0.00121	0.00279
48	water (I)	41	0.00124	0.00086	0.00230
14, 21, 28, 29, 30	water (I)	41	0.00094	0.00051	0.00138
45, 46, 47, 48, 49	water (I)	39	0.00070	0.00054	0.00132
14	coffee A (II)	41	0.00263 *	0.00017 *	0.00723 *
48	coffee A (II)	41	0.00157	0.00076	0.00287
14, 21, 28, 29, 30	coffee A (II)	40	0.00120	0.00074	0.00167
45, 46, 47, 48, 49	coffee A (II)	40	0.00077	0.00047	0.00120
14	coffee B (IV)	40	0.00259 *	0.00018 *	0.00727 *
48	coffee B (IV)	41	0.00146	0.00059	0.00268
14, 21, 28, 29, 30	coffee B (IV)	41	0.00115	0.00060	0.00188
45, 46, 47, 48, 49	coffee B (IV)	40	0.00077	0.00046	0.00153
14	reference (III)	41	0.00014	0.00008	0.00025
48	reference (III)	41	0.00032	0.00017	0.00062
14, 21, 28, 29, 30	reference (III)	41	0.00014	0.00008	0.00020
45, 46, 47, 48, 49	reference (III)	40	0.00023	0.00011	0.00036

**Table 4:** The statistical comparison results of medians ΔoxyHb for protocols I, II, III, and IV for channel 14 and channels 14, 21, 28, 29, and 30 for all participants.

	*p*-Values for									
Channel 14						Channels 14, 21, 28, 29, and 30				
vs.		Protocols				vs.	Protocols			
		III	I	II	IV		III	I	II	IV
Protocols	III		3.8 × 10^−16^	1.7 × 10^−16^	4.7 × 10^−16^	III		5.4 × 10^−15^	1.6 × 10^−15^	3.8 × 10^−15^
	I	3.8 × 10^−16^		0.12	0.16	I	5.4 × 10^−15^		0.12	0.18
	II	1.7 × 10^−16^	0.12		0.98	II	1.6 × 10^−15^	0.12		0.87
	IV	4.7 × 10^−16^	0.16	0.98		IV	3.8 × 10^−15^	0.18	0.87	

**Table 5:** The results of the statistical comparison of medians ΔoxyHb for protocols I, II, III, and IV for channel 48 and channels 45, 46, 47, 48, and 49 for all participants.

	*p*-Values for									
Channel 48						Channels 45, 46, 47, 48, and 49				
vs.		Protocols				vs.	Protocols			
		III	I	II	IV		III	I	II	IV
Protocols	III		2.3 × 10^−10^	9.7 × 10^−11^	1.5 × 10^−8^	III		9.0 × 10^−10^	5.4 × 10^−9^	5.2 × 10^−8^
	I	2.3 × 10^−10^		0.50	0.74	I	9.0 × 10^−10^		0.99	0.69
	II	9.7 × 10^−11^	0.50		0.80	II	5.4 × 10^−9^	0.99		0.68
	IV	1.5 × 10^−8^	0.74	0.80		IV	5.2 × 10^−8^	0.69	0.68	

**Table 6:** The statistical comparison results of medians ΔoxyHb for all analyzed channels for protocol I–III for all participants.

*p*-Values for Protocol I–III					
vs.		Channels			
		14	48	14, 21, 28–30	45–49
Channels	14		3.2 × 10^−3^	2.9 × 10^−5^	1.0 × 10^−6^
	48	3.2 × 10^−3^		0.37	0.04
	14, 21, 28–30	2.9 × 10^−5^	0.37		0.11
	45–49	1.0 × 10^−6^	0.04	0.11	

**Table 7:** The statistical comparison results of medians ΔoxyHb for all analyzed channels for protocol II–III for all participants.

*p*-Values for Protocol II–III					
vs.		Channels			
		14	48	14, 21, 28–30	45–49
Channels			1.3 × 10^−3^	2.0 × 10^−6^	5.5 × 10^−10^
	48	1.3 × 10^−3^		0.65	1.2 × 10^−2^
	14, 21, 28–30	2.0 × 10^−6^	0.65		6.5 × 10^−3^
	45–49	5.5 × 10^−10^	1.2 × 10^−2^	6.5 × 10^−3^	

**Table 8:** The results of the statistical comparison of medians ΔoxyHb for all analyzed channels for protocol IV–III for all participants.

*p*-Values for Protocol IV–III					
vs.		Channels			
		14	48	14, 21, 28–30	45–49
Channels	14		0.02	3.3 × 10^−5^	1.0 × 10^−6^
	48	0.02		0.44	0.04
	14, 21, 28–30	3.3 × 10^−5^	0.44		0.063
	45–49	1.0 × 10^−6^	0.04	0.063	

**Table 9:** The correlations between the amount of coffee consumed, the bitterness assessment solutions, and the signal height for individual channels. R—Spearman correlation coefficient, ACC—the amount of consumed coffee, bitterness—the bitterness assessment solutions.

		Variable Pairs		
		Bitterness & ΔoxyHb for Channels:	R	*p*-Value
Coffee (protocol)	A (II)	14	0.1	0.45
		48	−0.1	0.70
		14, 21, 28, 29, 30	−0.1	0.74
		45, 46, 47, 48, 49	−0.1	0.39
	B (IV)	14	0.4	<0.01
		48	0.2	0.19
		14, 21, 28, 29, 30	0.3	<0.05
		45, 46, 47, 48, 49	0.1	0.47
		**ACC & ΔoxyHb for channels:**	**R**	***p*** **-value**
Coffee (protocol)	A (II)	14	−0.2	0.18
		48	0.2	0.16
		14, 21, 28, 29, 30	0.004	0.97
		45, 46, 47, 48, 49	0.2	0.13
	B (IV)	14	−0.2	0.18
		48	0.02	0.90
		14, 21, 28, 29, 30	−0.2	0.14
		45, 46, 47, 48, 49	0.03	0.86

**Table 10:** The statistical comparison results of medians ΔoxyHb for all analyzed channels for protocol jaw movement—III for all participants.

Channels	Stimulation (Protocol)	n	ΔoxyHb [mmol/L]		
			Mdn	Q1	Q3
48	reference (III)	51	0.00036	0.00018	0.00067
45, 46, 47, 48, 49	reference (III)	50	0.00023	0.00011	0.00038
48	jaw movement	10	0.00026	0.00009	0.00057
45, 46, 47, 48, 49	jaw movement	10	0.00023	0.00012	0.00035

## Data Availability

Data supporting reported results can be found at https://doi.org/10.7910/DVN/SMPCFH accessed on 18 June 2024, Harvard Dataverse.
